# Direct detection of drug-resistant *Mycobacterium tuberculosis* using targeted next generation sequencing

**DOI:** 10.3389/fpubh.2023.1206056

**Published:** 2023-06-29

**Authors:** Shannon G. Murphy, Carol Smith, Pascal Lapierre, Joseph Shea, Kruthikaben Patel, Tanya A. Halse, Michelle Dickinson, Vincent Escuyer, Marie Claire Rowlinson, Kimberlee A. Musser

**Affiliations:** Wadsworth Center, New York State Department of Health, Albany, NY, United States

**Keywords:** mycobacterium, tuberculosis, drug susceptibility, resistance, targeted sequencing, nanopore

## Abstract

*Mycobacterium tuberculosis* complex (MTBC) infections are treated with combinations of antibiotics; however, these regimens are not as efficacious against multidrug and extensively drug resistant MTBC. Phenotypic (growth-based) drug susceptibility testing on slow growing bacteria like MTBC requires many weeks to months to complete, whereas sequencing-based approaches can predict drug resistance (DR) with reduced turnaround time. We sought to develop a multiplexed, targeted next generation sequencing (tNGS) assay that can predict DR and can be performed directly on clinical respiratory specimens. A multiplex PCR was designed to amplify a group of thirteen full-length genes and promoter regions with mutations known to be involved in resistance to first- and second-line MTBC drugs. Long-read amplicon libraries were sequenced with Oxford Nanopore Technologies platforms and high-confidence resistance mutations were identified in real-time using an in-house developed bioinformatics pipeline. Sensitivity, specificity, reproducibility, and accuracy of the tNGS assay was assessed as part of a clinical validation study. In total, tNGS was performed on 72 primary specimens and 55 MTBC-positive cultures and results were compared to clinical whole genome sequencing (WGS) performed on paired patient cultures. Complete or partial susceptibility profiles were generated from 82% of smear positive primary specimens and the resistance mutations identified by tNGS were 100% concordant with WGS. In addition to performing tNGS on primary clinical samples, this assay can be used to sequence MTBC cultures mixed with other mycobacterial species that would not yield WGS results. The assay can be effectively implemented in a clinical/diagnostic laboratory with a two to three day turnaround time and, even if batched weekly, tNGS results are available on average 15 days earlier than culture-derived WGS results. This study demonstrates that tNGS can reliably predict MTBC drug resistance directly from clinical specimens or cultures and provide critical information in a timely manner for the appropriate treatment of patients with DR tuberculosis.

## 1. Introduction

Tuberculosis (TB) continues to be a major contributor to global infectious disease deaths, with an estimated 10.6 million cases and 1.6 million deaths worldwide in 2021 (1). TB patients are treated with combination drug regimens; however, the emergence of increasingly drug-resistant forms of *Mycobacterium tuberculosis* complex (MTBC) in recent decades necessitates the use of alternative therapies (2). Currently, strategies for therapy are still mostly decided based on culture-based phenotypic drug susceptibility testing (DST); however, MTBC DST requires weeks or months to complete due to the slow growth rate of this organism (3–7). During this time, patients can be prescribed ineffective drug regimens, leading to treatment failure or the promotion of drug resistance (DR) (8). The potential for these negative patient outcomes underscores the need for quicker methods to detect DR TB (DR-TB).

Molecular and sequencing-based assays offer a faster alternative for profiling DR in slow-growing organisms like *M. tuberculosis*. Commercially available molecular methods include Xpert MTB/RIF (Cepheid, Sunnyvale, CA) (9) and the GenoType MTBDRplus line probe assay (Hain Lifescience Nehren, Germany) (10), which detect mutations within specific “hot spot” regions to predict DR. These rapid diagnostics, endorsed by World Health Organization (WHO), have contributed to improved global detection of DR, particularly for the first-line drug rifampin (11–14). These assays, however, may miss mutations outside of the targeted “hot spot” regions and incur false negative results (15, 16), or, in rare circumstances, silent or neutral mutations may incur false positive results for DR (17, 18).

Sequencing-based methods provide greater resolution of these loci. Assays developed for the detection of DR include pyrosequencing (19–22) and Sanger sequencing (23) of individual targets; however, these methods are typically limited to single-plex reactions analyzing limited sections of DR determining loci. NGS assays offer more comprehensive DR profiles by identifying novel and high confidence DR-associated mutations throughout the genome (24, 25). Whole genome sequencing (WGS) assays, such as the one implemented by Wadsworth Center, identify high-confidence mutations that allow accurate prediction of phenotypic DR (26). These assays provide comprehensive DR profiling and the bioinformatic analysis can be routinely updated to include new loci and mutations in accordance with national and global WHO databases (27). WGS results can be generated before phenotypic DST is available (7, 26, 28, 29); however, most clinically validated WGS assays are performed on MTBC-positive cultures that can require several weeks of incubation (30).

Targeted NGS (tNGS) assays can further reduce the time required for comprehensive DR profiling by amplifying numerous loci directly from clinical specimens. Several tNGS assays for DR profiling have been described in the literature, including laboratory-developed assays (31–35) and the commercially available Genoscreen Deeplex (36, 37) and Ion AmpliSeq (38). These assays vary in a number of ways including the selection and size of targets, how multiplexed the PCR reactions are, and the sequencing platforms employed, which include Illumina (31, 35, 36), Ion Torrent (32, 34, 38), and Oxford Nanopore Technologies (33, 39, 40).

In this paper, we describe the design, validation, and implementation of a tNGS assay for direct DR profiling on MTBC-positive clinical specimens at the Wadsworth Center. This assay includes a simplified set up with two multiplexed PCR amplification reactions that target thirteen full-length loci implicated in DR to first- and second-line MTBC antimicrobials. The assay was optimized for sequencing on an Oxford Nanopore Technologies platform, enabling real-time analysis, a two-to-three-day turnaround time with typically <2 h of sequencing time, and a cost of less than $80 per sample. In addition to performing tNGS on primary specimens, this assay was found to be accurate and generated susceptibility profiles comparable to those currently obtained with our existing WGS assay, which can only be performed on cultured isolates. These results demonstrate that tNGS-based assays can provide a reliable and cost-effective tool for early detection of DR-TB and should be considered for implementation in public health and clinical laboratories with MTBC testing needs and resources.

## 2. Materials and methods

### 2.1. Sample preparation

Samples submitted for mycobacterial testing were handled in a BSL-3 laboratory. Sterile tissue specimens (e.g., lung tissue, lymph node tissue) were homogenized in sterile saline. Respiratory specimens (e.g., sputum, bronchial washes, and bronchoalveolar lavages) underwent digestion and decontamination to optimize mycobacteria recovery. This procedure uses a 3.5% sodium hydroxide solution to dissolve mucus, lyse organic material, and inactivate other bacteria. Following incubation, the solution was neutralized, bacteria were concentrated by centrifugation, and the pellet was resuspended in a buffer. Processed samples were used to inoculate liquid cultures (MGIT 960, BACTEC) and underwent differential staining and smear microscopy. Aliquots for molecular testing were heat inactivated (80°C for 1 h) before handling in a BSL-2 laboratory.

### 2.2. Direct smear microscopy for acid-fast bacilli

Processed primary specimens were stained using the Ziehl-Neelsen Carbol Fuchsin method according to manufacturer instructions (Remel Inc., San Diego, CA) and examined under a microscope for the presence of Acid-Fast Bacilli (AFB). Samples positive for AFB were further categorized based on the number of AFB observed, with numerous defined as >9 AFB per high power field (HPF-1000X) (++++), moderate as 1**–**9 AFB per HPF (+++), few as 1**–**9 AFB per 10 HPF (++), and rare as 1**–**9 AFB per 300 HPF (+). Smear negative samples are defined as those with no AFB observed.

### 2.3. Real-time PCR for MTBC detection

DNA was extracted via mechanical lysis with FastPrep24 (MP Biomedicals, Solon, Ohio) and tested for *M. tuberculosis* complex (MTBC) DNA using previously described real-time PCR assay (41). This multiplexed assay includes a single-copy (ext-RD9) and multi-copy (IS*6110*) target for MTBC detection and a target for *Mycobacterium avium* complex detection (ITS). All specimens included in this study were positive for MTBC DNA via a real-time PCR.

### 2.4. Whole genome sequencing

Samples included in this study were analyzed using a NYS-validated WGS assay as previously described (26). Briefly, a manual DNA extraction utilizing InstaGene reagent (Bio-Rad Laboratories, Hercules, CA), mechanical lysis, and centrifugation was performed on heat-killed isolates identified as MTBC-positive. Concentration of DNA was assessed using Qubit DNA fluorometry (Thermo Fisher Scientific, Waltham, MA) and samples were prepared for Illumina sequencing on a MiSeq or NextSeq instrument (Illumina, San Diego, CA). Results were analyzed using a clinically validated in-house developed bioinformatics pipeline that identifies high-confidence and unknown/novel mutations (26).

### 2.5. DNA extraction and controls for tNGS

For tNGS, an automated lysis and purification-based DNA extraction method (EZ1 Virus DSP Kit, Qiagen) was used to minimize DNA shearing. On this platform, 100 μL of specimen was extracted and eluted in 60 μL. Each run included a positive and negative control, consisting of 100 μL of *Mycobacterium bovis* BCG MGIT positive culture and 100 μL of sterile molecular grade water, respectively. Both extraction controls were processed in parallel with clinicals specimens and serve as reagent and sequencing controls for the entire tNGS assay.

### 2.6. Primer design and PCR

Thirteen primer sets were designed to amplify full-length genes (*rpoB*, *katG*, *mabA*, *inhA*, *embB*, *gyrA*, *gyrB*, *ethA*, *rrs*, *rpsL*, and *pncA*) and/or promoter regions (*oxyR-ahpC*, *mabA-inhA*, *embC-A*, *pncA*, and *eis*) implicated in DR to first- and second-line MTBC antimicrobials, including rifampin, isoniazid, ethambutol, pyrazinamide, fluoroquinolones, ethionamide, streptomycin, and kanamycin/amikacin (Supplementary Table S1). Possible primer pairs were generated using Primer3 (42) and checked for *in silico* interactions with ThermoFisher’s Multiple Primer Analyzer Tool (ThermoFisher Scientific, Waltham, MA) (43). Primer sets were multiplexed into two PCR reactions referred to as “Pool A” and “Pool B” and primer concentrations were optimized to obtain balanced amplification of each target (Supplementary Table S2). Each 40 μL PCR reaction contained Long Amp Hot Start Taq Mastermix (New England Biolabs, Ipswich, MA), DMSO (5% final concentration), and 5 μL of template. PCR was run for 40 cycles (with a 3 min and 30 s extension time) according to manufacturer instructions. Amplicons were visualized via gel electrophoresis alongside a 1 kilobase ladder.

### 2.7. Library preparation for nanopore sequencing

**Figure 1:**
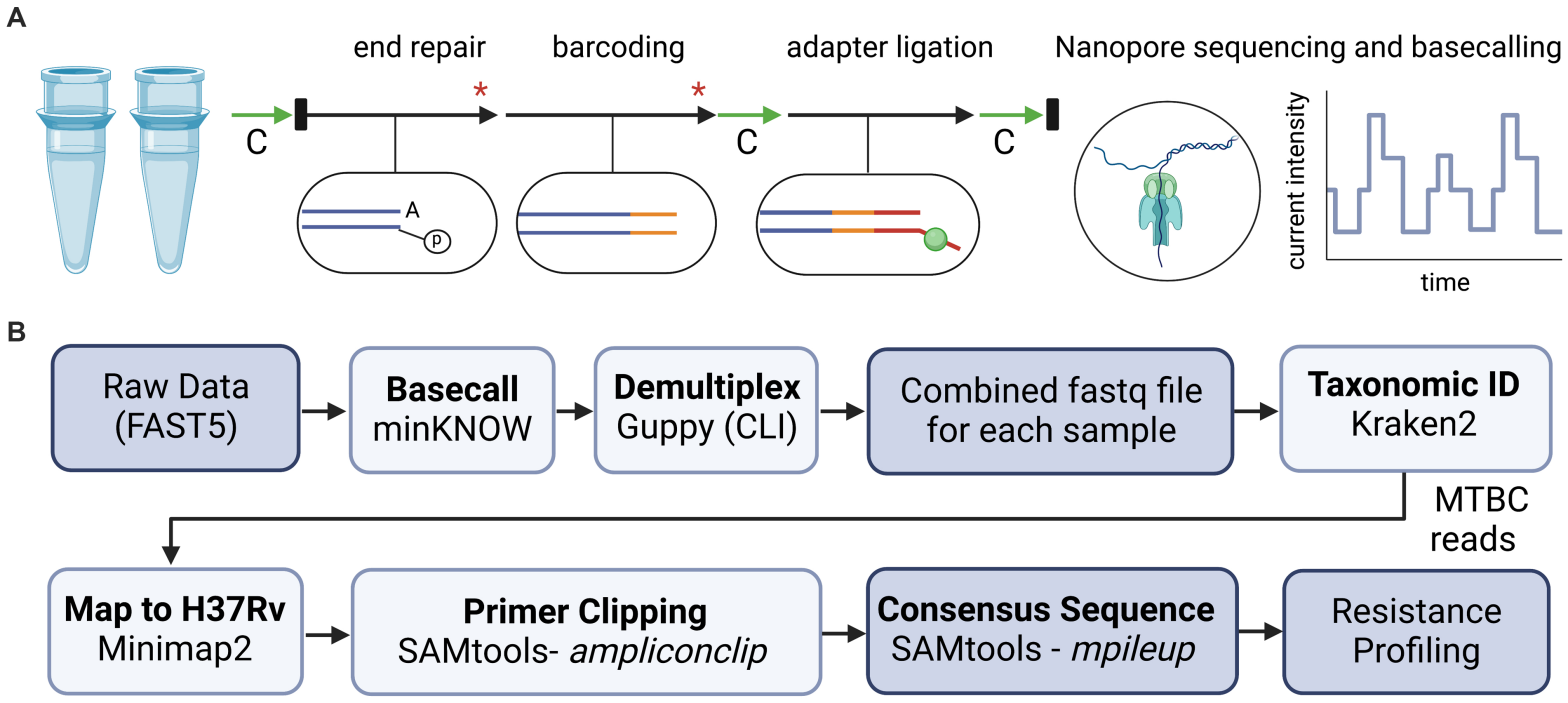
Overview of library preparation steps and bioinformatic analyses for tNGS nanopore sequencing. **(A)** For library preparation, two multiplex PCR reactions for each sample were combined and processed with AMPure bead-based clean-up steps (green arrows, “C”), enzymatic reactions (black arrows), dsDNA quantification via Qubit and normalization (black rectangles), and heat inactivation steps (red asterisk). **(B)** Bioinformatic tools used to analyze sequencing data and identify high confidence resistance mutations in MTBC. Diagrams created with BioRender.com.

PCR reactions for each sample were combined and prepared for sequencing using ligation-based reagents from Oxford Nanopore Technologies (ONT; Oxford, United Kingdom) and adapted protocols (44). An overview of library preparation steps is illustrated in Figure 1A. Briefly, amplicons were purified using AMPure XP (Beckman Coulter, Brea, CA) and quantified using a Qubit^™^ Flex Fluorometer (ThermoFisher Scientific, Waltham, MA). Samples were normalized for concentration prior to a two-step “spike-in” method for DNA end repair and barcode ligation (44). Barcoded products were purified using AMPure XP, followed by adapter ligation and a final AMPure XP clean-up. Final eluate concentrations were measured, samples were pooled in equal ratios, and the final library was diluted to a concentration of 35 ng/μL. A 12 μL volume of the library was loaded onto an R9 flow cell according to manufacturer instructions. The run was sequenced on either a MinION Mk1C or a GridION platform with high-accuracy base calling until approximately 50 k reads per sample were obtained. Flow cells were washed according to manufacturer instructions and reused only if the flow cell retained sufficient active pores (>450) and only with uniquely barcoded samples to limit potential cross-contamination.

### 2.8. Bioinformatic analysis

Oxford Nanopore Technologies sequencing data is analyzed in real-time using a custom bioinformatics pipeline (Figure 1B), akin to the NYS-validated WGS pipeline described in Shea et al. (26). The pipeline reads in each of the raw fastq files as they are generated using the MinKNOW interface on the instrument. Fastq files are demultiplexed using Guppy on a separate server via command line interface (CLI) with default parameters. Reads are combined into a final fastq file for each analyzed sample. The pipeline then assesses the taxonomic content of each file using Kraken2 (version 2.1.2) and the k2_standard_08gb_20220607 database (45). All non-*Mycobacterium* genus reads are filtered out for the rest of the downstream analyses. Reads are mapped to *Mycobacterium tuberculosis* H37Rv reference genome with minimap2 (version 2.24-r1122) (46) and amplicon primers sequences are hard-clipped from both ends using SAMtools (v 1.15.1) with ampliconclip (47). Finally, a high-quality consensus sequence is generated for each sample using SAMtools mpileup (47) with minimum mapping quality and base quality of Phred 30 and 12, respectively, and minimum depth of 10× and 60% allele agreement. Indels require 40× minimum depth and 55% allele agreement. In cases where indels are directly adjacent or inside homopolymeric regions of three or more identical bases, percent allele agreement is raised to 75%. If a position (variant or invariant) does not reach these requirements, it is assigned as ‘N’ on the consensus sequence. The pipeline identifies 86 high-confidence resistance mutations across the 51 positions listed in Supplementary Table S1, and notes novel/unknown mutations. The different cutoffs for single nucleotide polymorphisms (SNPs) and insertions/deletions (INDELs) were empirically determined by assessing the different allele frequencies (AF) over several runs and determining the best AF cutoff to avoid calling any false positive SNP or INDEL variants.

## 3. Results

### 3.1. Validation of tNGS for clinical use

To validate the tNGS assay for clinical use, we assessed sensitivity, reproducibility, specificity, and accuracy. To assess sensitivity of tNGS on respiratory specimens, a culture of the *M. tuberculosis* reference strain H37Rv (ATCC 25618) was serially diluted and spiked into processed negative sputa to determine the limit of detection (LOD). Average Ct-values for MTBC detection ranged from 24.2 to undetected and the concentration of *M. tuberculosis* in each PCR reaction ranged from 108 CFU to 0.00108 CFU (21,600 CFU to 0.216 CFU per mL). tNGS was performed on three replicate dilution series and sequenced to a total of approximately 80 k reads per sample. Quality control (QC) metrics were met for all targets (and corresponding drug classes) down to a lower detection limit of 0.108 CFU per reaction (Supplementary Table S3).

To measure reproducibility, three replicates of three smear positive specimens were processed in parallel (intra-assay) or on separate days (inter-assay). Results were concordant within and between runs, as shown in Supplementary Table S4. Specificity was tested against a panel of five organisms – including two mycobacteria (*Mycobacterium fortuitum* and *Mycobacterium abscessus*) and three other organisms common in sputa (*Klebsiella pneumoniae*, *Streptococcus pneumoniae*, and *Haemophilus influenzae*). No cross-reactivity was detected in this panel of organisms (Supplementary Table S5).

### 3.2. tNGS detection of drug resistance directly on respiratory specimens

To measure assay accuracy, tNGS was performed on a panel of 72 extracted primary specimens that were selected for their diverse mutations and drug resistance profiles. All specimens included in the panel were confirmed positive for MTBC DNA via real-time PCR. The panel consisted of 35 retrospective blinded samples and 37 prospective samples received over a period of 8 months (May 2022 to January 2023). The panel included predominantly sputa (*n* = 58, 81%) along with other respiratory specimens (i.e., bronchoalveolar lavages and bronchial washes) and rarer specimen types (i.e., lymph nodes and lung tissue). Specimens covered a range of MTBC concentrations (assessed by AFB smear and real-time PCR); most specimens included were AFB positive (*n* = 65, 90%), but five AFB negative samples and two untested samples were also included in the study (Supplementary Table S6).

The two multiplex PCR reactions were performed on the panel of specimens and amplification was confirmed by gel electrophoresis. Amplicons that could be visualized with ethidium bromide gel staining following PCR were present in 78% of the samples tested. High confidence resistance and unknown/novel mutations and DR profiles identified by tNGS were compared to those obtained from the NYS-validated WGS assay on isolates from the matched specimens. Profiles were defined according to CDC definitions: multidrug resistant (MDR; INH and RIF resistant), pre-extensively drug resistant (pre-XDR; INH, RIF, FQ), extensively drug resistant (XDR; INH, RIF, FQ, KAN/AMI). Resistance to other MTBC antimicrobials not meeting the criteria above is defined here as other mono- or poly-resistant (R). The results for each specimen are shown in Supplementary Table S6 and an aggregate summary is provided in Table 1. Of the MTBC-positive samples sequenced, tNGS correctly identified 44 pan-susceptible, 5 mono/poly-resistant, and 5 MDR, and 1 pre-XDR, and 1 XDR strain, all determined to have a DR profile identical to the WGS DR profile obtained from the culture isolate from the same case. At the mutation level, two tNGS reports identified additional unknown mutations in primary specimens that were not identified by WGS performed on cultured isolates. This raises the potential for tNGS to detect subpopulations in the primary clinical specimens. Overall, these results demonstrate that tNGS can accurately detect susceptible and DR forms of MTBC directly from primary specimens.

**Table 1:** Comparison of DR profiles identified by tNGS performed on primary specimens to WGS performed on matched MTBC-positive cultures.

		WGS (culture)				
		S	R	MDR	Pre-XDR	XDR
tNGS (Primary)	S	44	0	0	0	0
	R	0	5	0	0	0
	MDR	0	0	5	0	0
	Pre-XDR	0	0	0	1	0
	XDR	0	0	0	0	1
	Not sequenced	13	1	2	0	0
	Total	57	6	7	1	1

Profiles are categorized as pan-susceptible (S), mono- or poly-resistant (R), multidrug resistant (MDR), pre-extensively drug resistant (pre-XDR), and extensively drug resistant (XDR).

### 3.3. Primary specimen tNGS data quality

To evaluate data quality, samples were categorized based on the number of targets that met quality control thresholds defined by the analysis pipeline, either as complete susceptibility profiles (all 13 targets pass QC), partial susceptibility profiles (≥10 targets pass QC), or no profile (not sequenced). In the panel of 72 primary specimens, tNGS produced 68% complete profiles, 10% partial profiles, and the remaining 22% were not sequenced due to PCR failure. These results indicate that targeted sequencing data can be obtained direct from primary specimens, although there is some variability in data quality.

**Figure 2:**
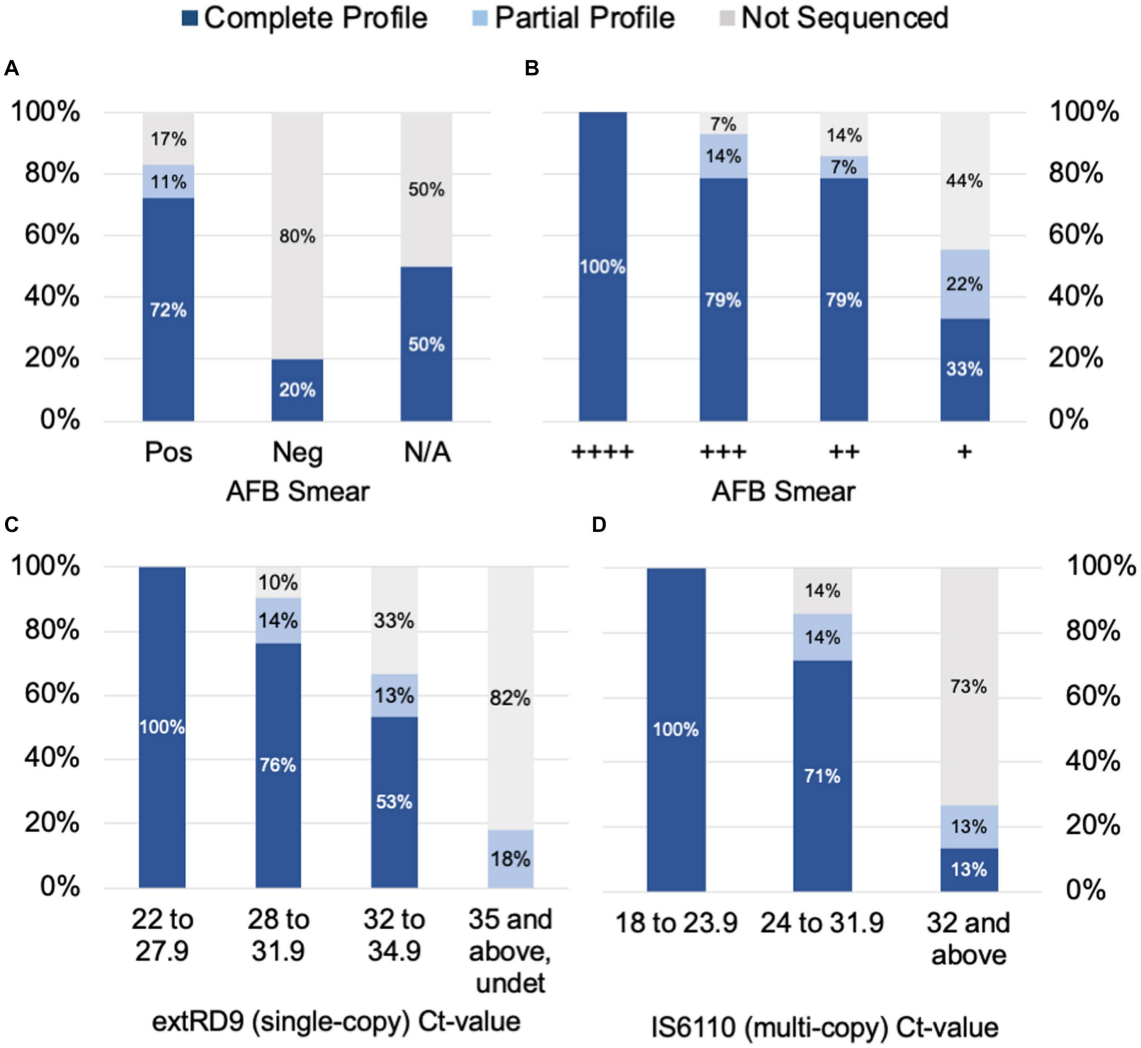
tNGS data completeness is arranged by AFB smear **(A)** and **(B)** tNGS data completeness is arranged by AFB smear and real-time PCR values for **(C)** a single-copy target RD9 and **(D)** multi-copy target IS*6110* for MTBC. Profiles are defined as complete (all 13 targets pass QC), partial (≥10 targets pass QC), or not sequenced.

To determine the factors that influence tNGS target failure and establish quality criteria for testing, the bacterial load in samples was estimated using AFB smear microscopy and MTBC real-time PCR Ct-values. Complete or partial profiles were obtained for 83% of the smear positive specimens tested (*n* = 65) (Figure 2A). Within the subset of smear positive samples, these percentages correlated with AFB smear results: 100% of samples with numerous AFB produced complete profiles, whereas complete or partial profiles were obtained for 93% of AFB moderate, 86% of AFB few, and 55% of AFB rare samples (Figure 2B). Of the five smear negative samples tested, only one specimen yielded a susceptibility profile; however, this sample had a low Ct-value uncharacteristic of a smear negative result (Supplementary Table S6). A smear could not be performed on several specimens due to specimen viscosity or insufficient volume for testing. These results indicate that target amplification is dependent on the quantity of AFB cells in the specimen and suggest that AFB smear-positive specimens are the most likely to yield complete susceptibility profiles.

Samples were also stratified by Ct-values derived from MTBC real-time PCR testing. Ct-values for the single-copy MTBC target (ext-RD9) ranged from 22.1 to 37.4 (or undetected) (Supplementary Table S6). Lower Ct-values yielded more complete tNGS sequencing results (Figure 2C); for values of 34.9 and below, 89% of samples yielded either complete or partial susceptibility profiles. In contrast, samples with Ct-values ≥35 were more prone to PCR failure (82%) and only two samples above this threshold produced a partial profile. Examination of Ct-values for IS*6110*, which is a multi-copy target and considered a more sensitive marker for MTBC detection, showed similar trends but with different ranges (Figure 2D). IS*6110* Ct-values ranged from 18.4 to 38.0 in the primary specimens tested (Supplementary Table S6). Samples with Ct-values ≤31.9 yielded either complete or partial susceptibility profiles (91%), whereas Ct-values ≥32 more were more prone to PCR failure (73%). These results indicate the quality of tNGS data is dependent on the amount of MTBC DNA present in the specimen and further suggests that quantification via real-time PCR may be used as a reliable metric for assessing sample quality for tNGS.

### 3.4. tNGS improves turnaround times

**Figure 3:**
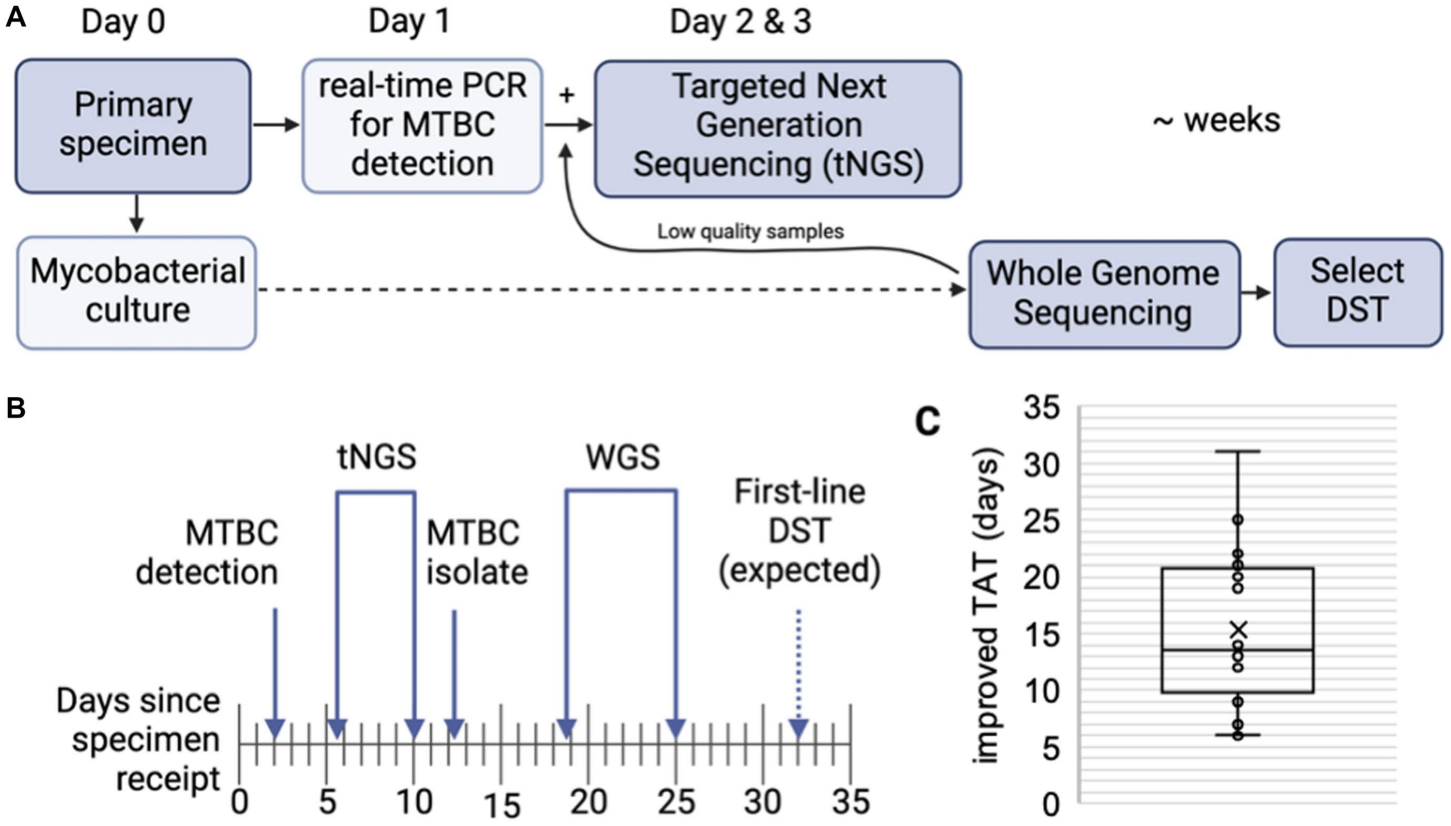
Turnaround times for MTBC molecular testing and sequencing. **(A)** MTBC testing algorithm at the Wadsworth Center. Processed specimens are used for mycobacterial culture. Heat killed aliquots are tested for MTBC DNA via real-time PCR and positive specimens are then referred to tNGS. When positive cultures are available, WGS is performed. Phenotypic drug-susceptibility testing (DST) is performed only if unknown/novel mutations or multidrug resistant strains are detected. **(B)** Timeline showing the average number of days required for MTBC DNA detection via real-time PCR, tNGS (from extraction to result), MTBC isolation, and WGS (from extraction to result) (*n* = 16). Note that tNGS and WGS assays are batched weekly and average turnaround times include non-business days (i.e., weekends). Estimated time for first-line DST results are indicated with a dashed arrow. **(C)** Turnaround time (TAT) improvements (in days) of direct tNGS compared to culture-derived WGS.

The ability of tNGS to generate comprehensive susceptibility profiles directly from a patient specimen has the potential to reduce turnaround times. A subset of 16 primary specimens with matched WGS results were used to calculate turnaround times; samples included in the analysis had tNGS performed as part of the routine testing algorithm (i.e., initiated within 1 week of MTBC detection) and yielded a positive MGIT culture suitable for WGS (Figure 3A). The average number of days required for MTBC detection via real-time PCR, tNGS (from extraction to result), MTBC isolation, and WGS (from extraction to result) are shown in Figure 3B.

Both tNGS and WGS samples were batched and run weekly. On average, tNGS results were available 10 days from sample receipt (or 7 business days if excluding weekends) (Figure 3B). This represents a 15 day reduction in turnaround time for tNGS versus WGS, with the improvement among samples ranging from 6 to 31 days (Figure 3C). Notably, one specimen included in this study was identified as XDR using tNGS and these results were available within 5 days from sample receipt, whereas culture-based WGS results were not available for an additional 3 weeks. These differences in turnaround time can largely be attributed to the incubation period required to obtain an AFB-positive culture and subsequent characterization (average 12.2 days); however, we also found that processing times – from sample extraction to final result – were shorter for nanopore-based tNGS (4.3 days or 2.3 business days) compared to Illumina-based WGS (6.3 days) in our current workflows (Figure 3B). These results demonstrate nanopore-based tNGS can offer comprehensive DR detection before MTBC isolates are available for WGS or culture-based DST.

### 3.5. tNGS on MTBC-positive cultures

tNGS may also provide additional utility for identifying high-confidence and unknown/novel mutations within MTBC-positive cultures. tNGS was performed on a panel of 55 MTBC-positive cultures, 21 of which were dual-positive for *M. avium* complex. Complete profiles were obtained for 100% isolates, whereas dual-positive cultures yielded either complete (86%) or partial profiles (14%) (Supplementary Table S7). Profiles were in 100% concordance with WGS (Table 2); however, two dual-positive samples did not have WGS available for comparison due to failure to obtain pure MTBC culture. These results demonstrate that tNGS can build comprehensive DR profiles from cultured material, even for mixed cultures that may not meet quality criteria for WGS analysis.

**Table 2:** Comparison of DR profiles identified by tNGS and WGS performed on MTBC-positive cultures.

		Whole Genome Sequencing (Culture)				
		S	R	MDR	pre-XDR	Not Sequenced
tNGS (Culture)	S	45	0	0	0	2
	R	0	3	0	0	0
	MDR	0	0	3	0	0
	pre-XDR	0	0	0	2	0
	Total	45	3	3	2	2

Profiles are categorized as pan-susceptible (S), mono or poly-resistant (R), multidrug resistant (MDR), and pre-extensively drug resistant (pre-XDR). Not sequenced indicates that a high-quality sample was not available for WGS analysis.

### 3.6. tNGS SNP-based lineage prediction

**Figure 4:**
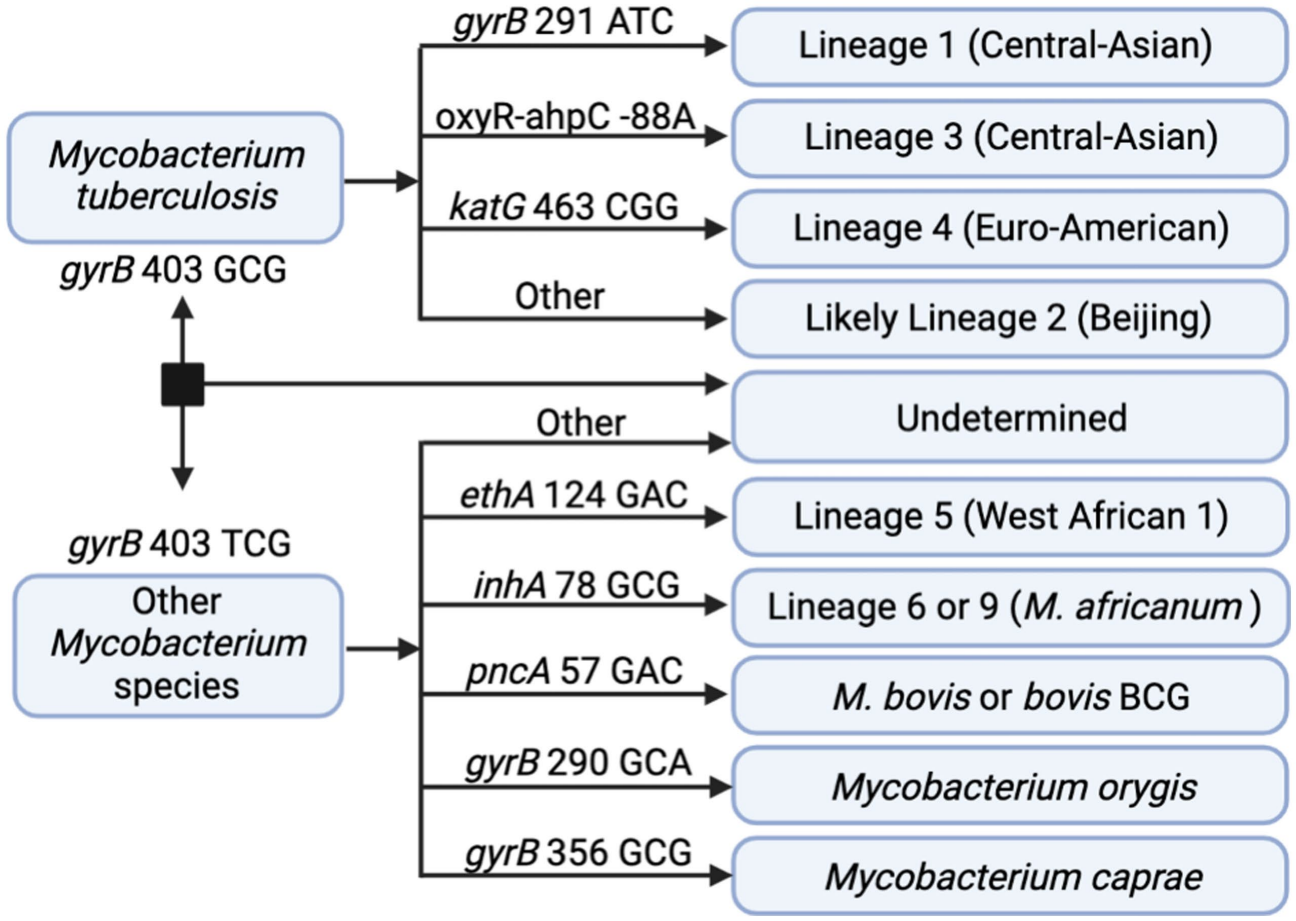
*In silico* SNP-based lineage classifications for MTBC. The SNP-based ID algorithm looks for unique SNPs in *gyrB*, *oxyR-ahpC*, *katG*, *ethA*, *inhA*, and *pncA* in the order shown below. Diagram created with BioRender.com.

In addition to DR profiling, tNGS data can also be used to identify the seven main phylogenetic MTBC lineages to provide supporting data during epidemiological investigations. *In silico* lineage predictions tools often utilize single-nucleotide polymorphisms (SNPs) to classify each lineage, but these SNP catalogs may vary (48). A SNP-based algorithm for lineage prediction was designed using the targets available in the tNGS assay (Figure 4). This algorithm initially relies on *gyrB* mutations that distinguish *M. tuberculosis* (*gyrB* 403 GCG mutation) from other *Mycobacterium* species (*gyrB* 403 TCG). For *M. tuberculosis* strains, the algorithm identifies markers for lineage 1 (*gyrB* 291 ATC), lineage 3 (*oxyR-ahpC*, −88 A), and lineage 4 (*katG* 463 CGG). MTBC strains not falling into these categories are classified as likely lineage 2; “likely” reflects the limitation that lineage 2 cannot be distinguished from the rarer lineage 7 with this set of loci. Other members of the MTBC are identified as lineage 5 (*ethA* 124 GAC), lineage 6 or 9 (*inhA* 78 GCG), *M. bovis* or *bovis* BCG (*pncA* 57 GAC), *M. orygis* (*gyrB* 290 GCA), or *M. caprae* (*gyrB* 356 GCG). Strains not fitting these criteria are not assigned with a lineage determination.

These SNP-based lineage predictions were performed on all samples included in this study (primary specimens and cultures) where both tNGS and WGS results were available (*n* = 109). This panel included lineages 1–4 and included one *M. bovis* BCG strain. Comparison of lineages derived *in silico* from tNGS and WGS are shown in Table 3. 98.2% of lineages were correctly identified by tNGS, 0.9% were undetermined due to target failure, and one lineage 4 strain (0.9%) was identified as “likely lineage 2” due to a heterogeneous SNP at *katG* 463. These results show that SNP-based lineage predictions are possible and highly accurate using a small number of loci.

**Table 3:** Concordance of *in silico* SNP-based lineage classifications from tNGS and WGS datasets.

		Lineage (WGS)				
		1	2	3	4	BCG
Lineage (tNGS)	Lineage 1	14	0	0	0	0
	Likely Lineage 2	0	31	0	1	0
	Lineage 3	0	0	11	0	0
	Lineage 4	0	0	0	50	0
	*M. bovis* or BCG	0	0	0	0	1
	Undetermined	1	0	0	0	0
	Total	15	31	11	51	1

### 3.7. Fiscal analysis of tNGS

The cost associated with nanopore-based tNGS is detailed in Table 4. The fixed cost per sample includes reagents for extraction ($12.17), PCR ($5.90) and library preparation ($37.21). The cost of gel electrophoresis is not included as this is considered an optional step. Some tNGS costs per sample are dependent on batch size; for example, each tNGS sequencing run requires $25.90 of reagents for flow cell priming, loading, and washing/storing regardless of the number of samples run. Other costs depend on flow cell reusage; we determined costs based on an average of 8 samples per run and up to three flow cell uses. Based on these estimates, the total estimated cost is $78.31 per sample. This analysis also does not include plastic consumables, technician time, instrumentation, or facility overhead as these factors may be facility specific and add to the overall price of the test. The cost per sample is similar to the cost of high-throughput WGS sequencing currently performed at the Wadsworth Center (49). These analyses indicate that tNGS assays can be cost-effective for implementation in diagnostic/clinical laboratories.

**Table 4:** Costs associated with tNGS.

tNGS steps	Total number of samples run on each flow cell			
	1 sample	8 samples	16 samples	24 samples
Extraction	$12.17	$97.36	$194.72	$292.08
PCR	$5.90	$47.22	$94.45	$141.67
Library preparation	$37.21	$297.69	$595.37	$836.40
Nanopore sequencing reagents^*^	$25.90 (1 run)	$25.90 (1 run)	$51.81 (2 runs)	$77.71 (3 runs)
Nanopore flow cell^**^	$475.00	$475.00	$475.00	$475.00
Total cost per sample	$556.19	$117.89	$88.21	$78.31

^*^Includes the cost of reagents for priming, loading, and washing/storing the flow cell. Cost is calculated per run and is independent of number of samples. ^**^Calculations assume up to 8 samples per run and up to three re-uses of the flow cell.

## 4. Discussion

### 4.1. tNGS is sensitive, scalable, and reliable for rapid prediction of drug resistance

tNGS represents a sensitive, reliable, and cost-effective method for detecting DR-TB direct from primary specimens in a clinical or diagnostic laboratory setting. This assay accurately identified diverse DR profiles – including MDR and XDR strains – with easier set-up than single-target assays and faster turnaround times than testing performed on cultured MTBC isolates, including WGS and phenotypic DST. This laboratory-developed tNGS assay represents an improvement to our current testing algorithm by offering comprehensive DR profiling shortly after TB diagnosis. Our study revealed a 15 day improvement in turnaround time compared to culture-based WGS, but additional experience will continue to inform tNGS implementation and improve the time to result.

### 4.2. tNGS assays require careful selection of targets and high-confidence mutations

tNGS assays require careful selection of targets and high confidence resistance mutations (50). Our assay targets full-length loci associated with resistance to first- and second-line MTBC antimicrobials and is consistent with other targeted assays (31–38), with some variation in number and size of loci included. In contrast to molecular beacon and line probe assays which focus just on hot spot regions, the assay described in this study examines full-length genes and promoter regions of many targets to allow for detection of rare and atypical resistance mutations. Although most smear positive specimens yielded complete susceptibility profiles, we found that longer targets (i.e., *embB*, *rpoB*) were more prone to low-coverage or amplification failure, resulting in partial susceptibility profiles. This observation suggests that sensitivity may be improved by splitting larger loci into multiple overlapping amplicons. One additional limitation of tNGS assays is that amplification may fail if strains carry mutations or deletions in primer binding regions, but these undetermined results will be further understood upon reflexing to WGS or culture-based DST in diagnostic testing algorithms (Figure 3A).

The accuracy of sequencing-based predictions for TB DR compared to phenotypic DST have been previously established for WGS (26); however, both tNGS and WGS assays require regular updates to keep pace with the emergence of new DR mutations. In the current Wadsworth Center testing algorithm, isolates with novel mutations undergo DST in order to characterize the potential impact of these mutations. A minimum of three isolates with paired phenotypic DST results or strong supporting literature are required to move novel mutations – initially reported as “unknowns” – to either a neutral or high confidence DR mutation list (26). Laboratories with smaller testing catalogs may refer to the WHO database (51) or other supporting literature to supplement their high confidence DR mutation list. tNGS assays may be updated with additional targets or multiplex pools to keep pace with emerging need, such as genotypic DST predictions for drugs included in the BPaLM/BPaL (bedaquiline, pretomanid, linezolid, moxifloxacin) regimens for treating MDR and XDR infections (52).

### 4.3. Considerations for implementing Oxford Nanopore sequencing

Special considerations and workflow adaptations are required for using Oxford Nanopore Technologies sequencing platforms. Raw data files from nanopore sequencing devices are basecalled into fastq files that are available for analysis. Newer versions of these basecalling algorithms continually provide better sequencing accuracy and, depending on the algorithm and flow cell version used, these accuracies can approach and even surpass Illumina-based platforms (53). These improvements demand greater processing requirements and thus can create lag times between sequencing and fastq file generation, thus, the use of graphics processing unit (GPU) or Cloud computing resources are highly recommended for basecalling and data post-processing (54, 55). Both the MinION Mk1C and GridION platforms from Oxford Nanopore Technologies were used in this study. While both platforms were able to take advantage of their GPUs for basecalling, we found that the compute power of GridION was able to perform high accuracy basecalling in real-time, enhancing turnaround times compared to the MinION Mk1C. The GridION, however, occupies a larger footprint in the laboratory and is less portable than the Mk1C for applications in resource-limited settings.

Future applications of this technology include detection of heterozygous positions, but this is currently limited by the accuracy of the sequencing data. Newer nanopore chemistries paired with more advanced basecalling algorithms show improved accuracy and potential for heterozygous detection (56). However, these updates to chemistry also necessitate frequent validation and verification. Thus, adoption of commercial products with longevity are critical for clinical implementation and use.

Consistent with other studies (40), we found that nanopore-based tNGS was cost-effective and comparable to current high-throughput WGS assays. Nevertheless, nanopore costs can vary widely depending on batch sizes and flow cell usage. To minimize cost, this validation study successfully obtained accurate tNGS data with re-used flow cells; however, we suggest using unique barcodes for each run to limit potential cross-contamination in clinical testing. Laboratories with lower testing volumes may consider combing multiple targeted assays onto one nanopore flow cell.

In conclusion, this study demonstrates the utility of a clinical tNGS assay as an early detection method for drug resistance direct from MTBC-positive specimens. This particular tNGS assay showed more than a two-week improvement in turnaround time compared to culture and WGS workflows at a similar cost. This method also offers additional utility for cultures that are low quality for WGS analysis due to mixed organisms or low MTBC DNA concentration. Early detection methods are an essential part of TB testing algorithms to ensure that patients are expeditiously placed on appropriate drug treatment regimens.

## Data availability statement

Illumina and MinION sequencing datasets are available in BioProject PRJNA766641 (https://www.ncbi.nlm.nih.gov/bioproject/?term=PRJNA766641) under the accession numbers listed in Supplementary Table S8.

## Author contributions

SM, CS, PL, and KM contributed to conception and design of the study. SM and CS optimized the targeted sequencing methods. SM, CS, KP, and JS collected data. JS identified mutations for *in silico* lineage prediction. PL developed bioinformatic pipelines and wrote sections of the manuscript. SM, PL, TH, MD, VE, M-CR, and KM provided feedback for implementation in the public health laboratory. SM wrote the first draft of the manuscript. All authors contributed to the article and approved the submitted version.

## Funding

This research project was supported by a grant from the National Institute of Allergy and Infectious Diseases (5R21AI13985602) and partially supported by NU50CK000516 (Epidemiology and Laboratory Capacity for Prevention and Control of Emerging Infectious Diseases in New York State).

## Conflict of interest

The authors declare that the research was conducted in the absence of any commercial or financial relationships that could be construed as a potential conflict of interest.

## Publisher’s note

All claims expressed in this article are solely those of the authors and do not necessarily represent those of their affiliated organizations, or those of the publisher, the editors and the reviewers. Any product that may be evaluated in this article, or claim that may be made by its manufacturer, is not guaranteed or endorsed by the publisher.
