# Supplementary material for: Direct detection of drug-resistant Mycobacterium tuberculosis using targeted next generation sequencing
Source: Front Public Health. 2023 Jun 29;11:1206056. doi: 10.3389/fpubh.2023.1206056 (PMC10340549; doi:10.3389/fpubh.2023.1206056)
Supplement: Supplementary file 1 [file Table_1.docx]

**Table S1**. List of high confidence mutations included in the bioinformatic analysis.

| **Antimicrobial (abbreviation)** | **Locus** | **Codon/NT position** | **Function** |
| --- | --- | --- | --- |
| Ethambutol (EMB) | *embB* | 306, 328, 354, 406, 497 | Coding |
| Ethambutol (EMB) | *embC-embA* promoter region | −12 | Non-coding |
| Fluoroquinolones (FQ) | *gyrA* | 74, 88, 90, 91, 94 | Coding |
| Fluoroquinolones (FQ) | *gyrB* | 446, 499 | Coding |
| Isoniazid (INH) | *katG* | 121, 191, 279, 315, 394, 525 | Coding |
| Isoniazid (INH) | *katG* | - | Stop codon |
| Isoniazid (INH) | *oxyR-ahpC* promoter region | −81 | Non-coding |
| Isoniazid (INH) | *mabA* | 203 | Coding |
| Isoniazid (INH) and Ethionamide (ETH) | *inhA* | 94 | Coding |
| Isoniazid (INH) | *mabA-inhA* promoter region | −17, −8 | Non-coding |
| Ethionamide (ETH) | *mabA-inhA* promoter region | −15 | Non-coding |
| Ethionamide (ETH) | *ethA* | 89 | Coding |
| Ethionamide (ETH) | *ethA* | - | Stop codon |
| Kanamycin (KAN) | *eis* promoter region | −10 and −37 | Non-coding |
| Kanamycin (KAN)/Amikacin (AMI) | *rrs* | 1400 | Non-coding |
| Pyrazinamide (PZA) | *pncA* | 47, 119, 163, 182 | Coding |
| Pyrazinamide (PZA) | *pncA* promoter region | −11 | Non-coding |
| Rifampin (RIF) | *rpoB* | *M. tuberculosis* numbering system: 170, 430, 432, 435, 441, 445, 450, 452, 491  *E. coli* numbering system: 146, 511, 513, 516, 522, 526, 531, 533, 572 | Coding |
| Streptomycin (STM) | *rpsL* | 43, 88 | Coding |
| Streptomycin (STM) | *rrs* | 512, 513, 516, 906 | Non-coding |
