# Supplementary material for: Direct detection of drug-resistant Mycobacterium tuberculosis using targeted next generation sequencing
Source: Front Public Health. 2023 Jun 29;11:1206056. doi: 10.3389/fpubh.2023.1206056 (PMC10340549; doi:10.3389/fpubh.2023.1206056)
Supplement: Supplementary file 2 [file Table_2.DOCX]

**Table S2**. Sequences, genomic positions, and concentrations of primers included in each multiplex PCR reaction.

| **Primer Name** | **Sequence (5'-3')** | **Genomic Position** | | **Amplicon Size (bp)** | **Final conc. (uM) of each primer** | | **Pool** |
| --- | --- | --- | --- | --- | --- | --- | --- |
|  |  | **Start** | **End** |  |  |  |  |
| tNGS-gyrA-F | CGCCGCTGTACAAACTCAAG | 6936 | 6955 | 2978 | | 0.33 µM | A |
| tNGS-gyrA-R | CCCGACTCCTAACACTCGTA | 9894 | 9913 |  |  |  |  |
| tNGS-mabA-inhA-F | AATGGAAATCGACTGGTCAGGT | 1672917 | 1672938 | 2902 | | 0.14 µM |  |
| tNGS-mabA-inhA-R | CTTGTAATCGCAACTCGTGGTC | 1675797 | 1675818 |  |  |  |  |
| tNGS-rpoB-F | CTCCTCTAAGGGCTCTCGTT | 759758 | 759777 | 3662 | | 0.5 µM |  |
| tNGS-rpoB-R | GCGGTAGCAAGACCGATG | 763402 | 763419 |  |  |  |  |
| tNGS-ethA-F | GACGGCCTCGACATTACGTT | 4327488 | 4327507 | 1828 | | 0.09 µM |  |
| tNGS-ethA-R | TTGCTGAAGTCGGTGCTCTC | 4325642 | 4325661 |  |  |  |  |
| tNGS-katG-F | GGACCATAACGGCTTCCTGT | 2156226 | 2156245 | 2503 | | 0.36 µM |  |
| tNGS-katG-R | CATTTCGGCGCCCTTTCTC | 2153706 | 2153724 |  |  |  |  |
| tNGS-eis-F | CCGGTACTTGCTCTGCAC | 2715529 | 2715546 | 1329 | | 0.12 µM |  |
| tNGS-eis-R | CGAAGCAGCTGGGAATCTTT | 2714182 | 2714201 |  |  |  |  |
| tNGS-embB-F | GATCGGTGGAGCAGTACCA | 4246396 | 4246414 | 3636 | | 0.8 µM | B |
| tNGS-embB-R | GCTGAGGAGACTTTTGTGGACTG | 4250009 | 4250031 |  |  |  |  |
| tNGS-gyrB-F | GCGCGGTTAGATGGGTAAA | 5113 | 5131 | 2380 | | 0.28 µM |  |
| tNGS-gyrB-R | AAGCCGGAATCGAACATTG | 7474 | 7492 |  |  |  |  |
| tNGS-rrs-F | AGATCGAACGGGTATGCTGTTA | 1471555 | 1471576 | 1927 | | 0.13 µM |  |
| tNGS-rrs-R | GGCCAACTTTGTTGTCATGC | 1473462 | 1473481 |  |  |  |  |
| tNGS-embC-embA-F | ACAGCGGTTGACGCCTTACT | 4243042 | 4243061 | 1652 | | 0.09 µM |  |
| tNGS-embC-embA-R | GTACTTGATGCGTGCCGATT | 4244674 | 4244693 |  |  |  |  |
| tNGS-oxyR-ahpC-F | GTGGTTGCTATGCGATCAGG | 2725553 | 2725572 | 1138 | | 0.08 µM |  |
| tNGS-oxyR-ahpC-R | GTCGAGCACTCGCAGTACCT | 2726671 | 2726690 |  |  |  |  |
| tNGS-rpsL-F | AATACACCGCTGCGACTAGG | 781064 | 781083 | 939 | | 0.08 µM |  |
| tNGS-rpsL-R | GACCAACTGCGATCCGTAGA | 781983 | 782002 |  |  |  |  |
| tNGS-pncA-F | GACGGATTTGTCGCTCACTA | 2289391 | 2289410 | 876 | | 0.06 µM |  |
| tNGS-pncA-R | CCCGATGAAGGTGTCGTAGAA | 2288496 | 2288516 |  |  |  |  |
