# Supplementary material for: Direct detection of drug-resistant Mycobacterium tuberculosis using targeted next generation sequencing
Source: Front Public Health. 2023 Jun 29;11:1206056. doi: 10.3389/fpubh.2023.1206056 (PMC10340549; doi:10.3389/fpubh.2023.1206056)
Supplement: Supplementary file 3 [file Table_3.docx]

**Table S3.** Targeted NGS sensitivity in spiked sputum.

| **H37Rv** | | **Median Ct value** ^1^ | | **tNGS Results** | | | |
| --- | --- | --- | --- | --- | --- | --- | --- |
| CFU  per 1 mL | CFU  per 5µL | **ExtRD9**  (single copy) | **IS6110**  (multi-copy) | **QC**  (#1) | **QC**  (#2) | **QC**  (#3) | **Taxonomic Match ^4^** |
| **21,600** | **108** | 24.2 | 20.7 | Pass**^2^** | Pass**^2^** | Pass**^2^** | *M. tuberculosis* |
| **2,160** | **10.8** | 27.3 | 23.8 | Pass**^2^** | Pass**^2^** | Pass**^2^** | *M. tuberculosis* |
| **216** | **1.08** | 30.9 | 26.8 | Pass**^2^** | Pass**^2^** | Pass**^2^** | *M. tuberculosis* |
| **21.6** | **0.108** | 41.2 | 30.0 | Pass**^2^** | Pass**^2^** | Pass**^2^** | *M. tuberculosis* |
| 2.16 | 0.0108 | undetected | 39.3 | Fail (10) **^3^** | Fail (13) **^3^** | Fail (13) **^3^** | *Streptomyces* sp. |
| 0.216 | 0.00108 | undetected | undetected | Fail (13) **^3^** | Fail (13) **^3^** | Fail (7)**^3^** | *Streptomyces* sp*.* |

1. Ct-values for *M. tuberculosis* complex single-copy target (RD9) and multi-copy target (IS6110) represent the median of three replicates.
2. “Pass” indicates that all 13 targets met the minimum quality control (QC) requirements.
3. “Fail” indicates that multiple targets did not meet QC metrics. The number of targets that failed are indicated in parentheses.
4. Top taxonomic match was identified using Kraken, which assigns taxonomic labels to short DNA sequences (Wood *et al*., 2019).
