# Supplementary material for: Direct detection of drug-resistant Mycobacterium tuberculosis using targeted next generation sequencing
Source: Front Public Health. 2023 Jun 29;11:1206056. doi: 10.3389/fpubh.2023.1206056 (PMC10340549; doi:10.3389/fpubh.2023.1206056)
Supplement: Supplementary file 4 [file Table_4.DOCX]

**Table S4**. **tNGS was performed on three clinical specimens on three separate days or in triplicate on the same run to assess inter-assay and intra-assay reproducibility, respectively.** Results indicate susceptibilities for 8 antimicrobials: rifampin, isoniazid, pyrazinamide, ethambutol, streptomycin, kanamycin/amikacin, fluoroquinolones, and ethionamide. Strains are considered “susceptible” for each drug unless otherwise indicated in the table below as resistant (**bold**) or unknown (-unk).

| **Sample** | | **tNGS Results** | | | **WGS Results** | |
| --- | --- | --- | --- | --- | --- | --- |
| **ID** | **AFB ^1^** | **Date Performed** | **High Confidence Mutations ^2^**  **[Unknown mutations]** | **Susceptibility Profile ^3^** | **High Confidence Mutations ^2^**  **[Unknown mutations]** | **Susceptibility Profile ^3^** |
| 35 | ++++ | 3/15/22 | *rpoB* Ser531Leu  *mabA* Leu203Leu  *embB* Met306Ile  *rpsL* Lys43Arg | **RIF**  **INH/ETH**  **EMB**  **SM** | *rpoB* Ser531Leu  *mabA* Leu203Leu  *embB* Met306Ile  *rpsL* Lys43Arg | **RIF**  **INH/ETH**  **EMB**  **SM** |
|  |  | 3/22/22^4^ | *rpoB* Ser531Leu  *mabA* Leu203Leu  *embB* Met306Ile  *rpsL* Lys43Arg | **RIF**  **INH/ETH**  **EMB**  **SM** |  |  |
|  |  | 4/27/22 | *rpoB* Ser531Leu  *mabA* Leu203Leu  *embB* Met306Ile  *rpsL* Lys43Arg | **RIF**  **INH/ETH**  **EMB**  **SM** |  |  |
| 3 | +++ | 3/15/22 | None | Pan-susceptible | None | Pan-susceptible |
|  |  | 3/16/22^4^ | None | Pan-susceptible |  |  |
|  |  | 4/27/22 | None | Pan-susceptible |  |  |
| 25 | ++ | 3/15/22 | *rpoB* Ser531Leu  *katG* Ser315Thr  *rpsL* Lys43Arg  *eis* G(-10)A  *ethA* Tyr140STOP  [*embB* Tyr319Cys] | **RIF**  **INH**  **SM**  **KAN/AMI**  **ETH**  EMB-unk | *rpoB* Ser531Leu  *katG* Ser315Thr  *rpsL* Lys43Arg  *eis* G(-10)A  *ethA* Tyr140STOP  [*embB* Tyr319Cys] | **RIF**  **INH**  **SM**  **KAN/AMI**  **ETH**  EMB-unk |
|  |  | 3/22/22^4^ | *rpoB* Ser531Leu  *katG* Ser315Thr  *rpsL* Lys43Arg  *eis* G(-10)A  *ethA* Tyr140STOP  [*embB* Tyr319Cys] | **RIF**  **INH**  **SM**  **KAN/AMI**  **ETH**  EMB-unk |  |  |
|  |  | 4/27/22 | *rpoB* Ser531Leu  *katG* Ser315Thr  *rpsL* Lys43Arg  *eis* G(-10)A  *ethA* Tyr140STOP  [*embB* Tyr319Cys] | **RIF**  **INH**  **SM**  **KAN/AMI**  **ETH**  EMB-unk |  |  |
| 27 | ++++ | 3/15/22 | *rpoB* Asp516Tyr  *katG* Ser315Thr  *rpsL* Lys43Arg  *gyrA* Asp94Ala | **RIF**  **INH**  **SM**  **FQ** | *rpoB* Asp516Tyr  *katG* Ser315Thr  *rpsL* Lys43Arg  *gyrA* Asp94Ala | **RIF**  **INH**  **SM**  **FQ** |
|  |  | 3/15/22 | *rpoB* Asp516Tyr  *katG* Ser315Thr  *rpsL* Lys43Arg  *gyrA* Asp94Ala | **RIF**  **INH**  **SM**  **FQ** |  |  |
|  |  | 3/15/22 | *rpoB* Asp516Tyr  *katG* Ser315Thr  *rpsL* Lys43Arg  *gyrA* Asp94Ala | **RIF**  **INH**  **SM**  **FQ** |  |  |
| 14 | +++ | 3/15/22 | None | Pan-susceptible | None | Pan-susceptible |
|  |  | 3/15/22 | None | Pan-susceptible |  |  |
|  |  | 3/15/22 | None | Pan-susceptible |  |  |
| 20 | ++ | 3/15/22 | *rpoB* Ser531Phe  *katG* Ser315Thr  *embB* Met306Val  *rpsL* Lys43Arg  *eis* G(-37)T | **RIF**  **INH**  **EMB**  **SM**  **KAN/AMI** | *rpoB* Ser531Phe  *katG* Ser315Thr  *embB* Met306Val  *rpsL* Lys43Arg  *eis* G(-37)T | **RIF**  **INH**  **EMB**  **SM**  **KAN/AMI** |
|  |  | 3/15/22 | *rpoB* Ser531Phe  *katG* Ser315Thr  *embB* Met306Val  *rpsL* Lys43Arg  *eis* G(-37)T | **RIF**  **INH**  **EMB**  **SM**  **KAN/AMI** |  |  |
|  |  | 3/15/22 | *rpoB* Ser531Phe  *katG* Ser315Thr  *embB* Met306Val  *rpsL* Lys43Arg  *eis* G(-37)T | **RIF**  **INH**  **EMB**  **SM**  **KAN/AMI** |  |  |

1. Acid Fast Bacilli (**AFB**) observed with microscopy (++++, numerous; +++, moderate; ++, few)
2. High confidence resistance mutations detected. A full list can be found in **Supplemental Table 1.**
3. **RIF**, rifampin; **INH**, isoniazid; **PZA**, pyrazinamide; **EMB**, ethambutol; **FQ**, fluoroquinolones; **SM**, streptomycin; **KAN**, kanamycin; **AMI**, amikacin; **ETH**, ethionamide; -**res**, resistant; -**unk**; susceptibility unknown.

Replicate was performed as part of the blinded accuracy panel.
