# Supplementary material for: Direct detection of drug-resistant Mycobacterium tuberculosis using targeted next generation sequencing
Source: Front Public Health. 2023 Jun 29;11:1206056. doi: 10.3389/fpubh.2023.1206056 (PMC10340549; doi:10.3389/fpubh.2023.1206056)
Supplement: Supplementary file 5 [file Table_5.DOCX]

**Table S5.** tNGS was performed on 1 McFarland lysates of *M. tuberculosis* H37Rv and five non-target organisms to test assay specificity.

| **Organism** | **Strain ID** | **tNGS Results** | |
| --- | --- | --- | --- |
|  |  | **QC** ^1^ | **Top Taxonomic Match ^2^** |
| *Mycobacterium tuberculosis* H37Rv | ATCC 25177 | Pass | *Mycobacterium tuberculosis* |
| *Mycobacterium abscessus* | ATCC 23006 | Fail | *Mycobacteroides abscessus* |
| *Mycobacterium fortuitum* | ATCC 6841 | Fail | *Mycolicibacterium fortuitum* |
| *Haemophilus influenzae* (serotype A) | ATCC 9006 | Fail | *Haemophilus influenzae* |
| *Klebsiella pneumoniae* | ATCC BAA 1706 | Fail | *Klebsiella pneumoniae* |
| *Streptococcus pneumoniae* (serotype 1) | CDC SP-80 | Fail | *Streptococcus pneumoniae* |

^1^ Quality Control **(QC)** results are summarized for all 13 targets.

^2^ Top taxonomic match was identified using Kraken, which assigns taxonomic labels to short DNA sequences (Wood *et al*., 2019).
