# Supplementary material for: Direct detection of drug-resistant Mycobacterium tuberculosis using targeted next generation sequencing
Source: Front Public Health. 2023 Jun 29;11:1206056. doi: 10.3389/fpubh.2023.1206056 (PMC10340549; doi:10.3389/fpubh.2023.1206056)
Supplement: Supplementary file 6 [file Table_6.DOCX]

**Table S6. Comparison of resistance profiles obtained from tNGS (performed on 72 MTBC-positive primary specimens) with results obtained from WGS (performed on a paired culture isolate).** Results indicate susceptibilities to 8 antimicrobials: rifampin, isoniazid, pyrazinamide, ethambutol, streptomycin, kanamycin/amikacin, fluoroquinolones, and ethionamide. Strains are considered “susceptible” for each drug unless otherwise indicated in the table below as resistant (**bold**), unknown (-unk), or could not determine (-cnd). Profiles are categorized as pan-susceptible (**S**), mono- or poly-resistant (**R**), multidrug resistant (**MDR**), pre-extensively drug resistant (**pre-XDR**), and extensively drug resistant (**XDR**). Discrepancies are noted with asterisks and detailed below.

| **Specimen** | | | **Real-time PCR** | | **tNGS Results** | | | **WGS Results** | **Profile** |
| --- | --- | --- | --- | --- | --- | --- | --- | --- | --- |
| **ID** | **Source^1^** | **AFB^2^** | **IIS6110^3^** | **ExtRD9^3^** | **QC^4^** | **High Confidence Mutations^5^**  **[Unknown]** | **Susceptibility Profile^6^** | **Susceptibility Profile^6^** |  |
| 1 | BRW | + | 27.1 | 28.9 | pass | None | pan-susceptible | pan-susceptible | S |
| 2 | SPT | ++ | 24.6 | 28.2 | pass | *rpoB* His526Tyr | **RIF** | **RIF**  SM-cnd | **R** |
| 3 | SPT | +++ | 25.1 | 28.1 | pass | None | pan-susceptible | pan-susceptible | S |
| 4 | SPT | +++ | 28.0 | 28.4 | pass | [*katG* Tyr597Cys] | INH-unk | INH-unk | S |
| 5 | SPT | +++ | 25.1 | 28.4 | pass | None | pan-susceptible | pan-susceptible | S |
| 6 | SPT | ++ | 27.2 | 31.0 | pass | [*pncA* C(-32) A] | PZA-unk | PZA-unk | S |
| 7 | BRW | + | 32.2 | 34.8 | pass | None | pan-susceptible | pan-susceptible | S |
| 8 | SPT | + | 33.1 | 37.2 | n/a | n/a | n/a | pan-susceptible SM-cnd | S |
| 9 | SPT | ++ | 27.9 | 32.0 | pass | *katG* Ser315Thr  *rpsL* Lys88Gln  [*embC-A* promoter C(-59)A] | **INH**  **SM**  EMB-unk | **INH**  **SM**  EMB-unk | **R** |
| 10 | BRW | ++++ | 23.8 | 26.9 | pass | None | pan-susceptible | pan-susceptible | S |
| 11 | SPT | + | 36.7 | 36.0 | n/a | n/a | n/a | pan-susceptible | S |
| 12 | BAL | ++ | 24.8 | 28.9 | n/a | n/a | n/a | pan-susceptible | S |
| 13 | BAL | ++ | 30.6 | 33.7 | pass | [*eis* G(-35)T] | AMI/KAN-unk | AMI/KAN-unk | S |
| 14 | SPT | +++ | 20.5 | 23.7 | pass | None | pan-susceptible | pan-susceptible | S |
| 15 | SPT | +++ | 25.5 | 28.7 | pass | None | pan-susceptible | pan-susceptible | S |
| 16 | SPT | ++++ | 23.7 | 27.2 | pass | None | pan-susceptible | pan-susceptible | S |
| 17 | SPT | + | 35.7 | 35.3 | n/a | n/a | n/a | pan-susceptible | S |
| 18 | SPT | + | 31.4 | 34.4 | pass | None | pan-susceptible | pan-susceptible | S |
| 19 | SPT | + | 34.8 | 37.3 | n/a | n/a | n/a | pan-susceptible | S |
| 20 | SPT | ++ | 26.7 | 30.7 | pass | *rpoB* Ser531Phe  *katG* Ser315Thr  *embB* Met306Val  *rpsL* Lys43Arg  *eis* G(-37)T | **RIF**  **INH**  **EMB**  **SM**  **AMI/KAN** | **RIF**  **INH**  **EMB**  **SM**  **AMI/KAN** | **MDR** |
| 21 | SPT | ++ | 28.2 | 31.9 | pass | [*embB* Phe440Ser] | EMB-unk | EMB-unk | S |
| 22 | SPT | + | 31.2 | 34.7 | n/a | n/a | n/a | pan-susceptible | S |
| 23 | SPT | ++ | 33.7 | 34.7 | pass | None | pan-susceptible | pan-susceptible | S |
| 24 | SPT | ++++ | 19.6 | 22.9 | pass | None | pan-susceptible | pan-susceptible | S |
| 25 | SPT | ++ | 27.9 | 32.6 | pass | *rpoB* Ser531Leu  *katG* Ser315Thr  *rpsL* Lys43Arg  *eis* (G-10)A  *ethA* Tyr140STOP  [*embB* Tyr319Cys] | **RIF**  **INH**  **SM**  **AMI/KAN**  **ETH**  EMB-unk | **RIF**  **INH**  **SM**  **AMI/KAN**  **ETH**  EMB-unk | **MDR** |
| 26 | SPT | ++++ | 26.0 | 28.4 | pass | None | pan-susceptible | pan-susceptible | S |
| 27 | SPT | ++++ | 20.5 | 24.2 | pass | *rpoB* Asp516Tyr  *katG* Ser315Thr  *rpsL* Lys43Arg  *gyrA* Asp94Ala | **RIF**  **INH**  **SM**  **FQ** | **RIF**  **INH**  **SM**  **FQ** | **PRE-XDR** |
| 28 | SPT | + | 28.0 | 31.3 | pass | *rpoB* Ser531Leu | **RIF** | **RIF** | **R** |
| 29 | BRW | + | 28.5 | 31.8 | *rpoB, katG* | None | pan-susceptible  INH/RIF-cnd | pan-susceptible | S |
| 30 | SPT | + | 30.3 | 30.4 | Pass | None | pan-susceptible | pan-susceptible | S |
| 31 | SPT | ++ | 25.5 | 29.6 | Pass | None | pan-susceptible | pan-susceptible | S |
| 32 | BAL | + | 31.4 | 36.8 | *embB* | *rpoB* Leu533Pro | **RIF**  EMB-cnd | **RIF** | **R** |
| 33 | SPT | ++++ | 22.3 | 25.2 | Pass | None | pan-susceptible | pan-susceptible | S |
| 34 | SPT | + | 30.4 | 32.7 | Pass | None | pan-susceptible | pan-susceptible | S |
| 35 | SPT | ++++ | 22.6 | 26.5 | Pass | *rpoB* Ser531Leu  *mabA* Leu203Leu  *embB* Met3016Ile  *rpsL* Lys43Arg | **RIF**  **INH/ETH**  **EMB**  **SM** | **RIF**  **INH/ETH**  **EMB**  **SM** | **MDR** |
| 36 | SPT | + | 32.4 | 36.2 | n/a | n/a | n/a | pan-susceptible | S |
| 37 | SPT | - | 36.4 | undet | n/a | n/a | n/a | pan-susceptible | S |
| 38 | UNK | + | 25.5 | 29.6 | *rpoB, embB, gyrA* | [katG Lys639Glu]* | INH-unk*  RIF/EMB/FQ-cnd | pan-susceptible | S |
| 39 | SPT | ++++ | 21.4 | 24.2 | pass | None | pan-susceptible | pan-susceptible | S |
| 40 | SPT | +++ | 26.5 | 27.6 | pass | None | pan-susceptible | pan-susceptible | S |
| 41 | PLF | n/a | 32.3 | undet | n/a | n/a | n/a | pan-susceptible | S |
| 42 | SPT | +++ | 32.4 | 34.3 | *rpoB, embB* | None | pan-susceptible RIF/EMB-cnd | pan-susceptible | S |
| 43 | SPT | + | 29.4 | 33.5 | *embB* | [ethA Val104Met]  [*mabA* Ala43Val]*  [*rpoB* Glu330Lys]*  [*rpoB* Arg556His]* | pan-susceptible ETH-unk  RIF-unk*  INH-unk*  (EMB-cnd) | Pan-susceptible  ETH-unk | S |
| 44 | SPT | +++ | 23.9 | 27.3 | pass | None | pan-susceptible | pan-susceptible | S |
| 45 | SPT | +++ | 24.1 | 26.0 | pass | None | pan-susceptible | pan-susceptible (INH-cnd) | S |
| 46 | SPT | ++++ | 23.6 | 26.8 | pass | *rpoB* Asp516val  *katG* Ser315Thr  [*pncA* Gln10Arg]  *embB* Tyr319Ser | **RIF**  **INH**  PZA-unk  EMB-unk | **RIF**  **INH**  PZA-unk  EMB-unk | **MDR** |
| 47 | UNK | n/a | 23.3 | 23.7 | pass | *pncA* His57Asp | **PZA** | **PZA** | **R** |
| 48 | BAL | - | 32.2 | 34.7 | n/a | n/a | n/a | **RIF, INH, SM** | **MDR** |
| 49 | TISS | ++++ | 19.7 | 24.2 | pass | None | pan-susceptible | pan-susceptible | S |
| 50 | SPT | - | 34.6 | 36.9 | n/a | n/a | n/a | **RIF, INH, SM, ETH** | **MDR** |
| 51 | SPT | +++ | 29.9 | 29.2 | pass | None | pan-susceptible | pan-susceptible | S |
| 52 | SPT | + | 30.7 | 33.0 | n/a | n/a | n/a | **INH** | **R** |
| 53 | SPT | - | 38.0 | 37.4 | n/a | n/a | n/a | pan-susceptible | S |
| 54 | SPT | ++++ | 21.4 | 24.1 | pass | None | pan-susceptible | pan-susceptible | S |
| 55 | SPT | +++ | 21.9 | 25.0 | pass | None | pan-susceptible | pan-susceptible | S |
| 56 | SPT | ++++ | 18.4 | 22.1 | pass | None | pan-susceptible | pan-susceptible | S |
| 57 | BAL | +++ | 25.4 | 28.1 | n/a | n/a | n/a | pan-susceptible | S |
| 58 | SPT | ++++ | 23.7 | 27.6 | pass | None | pan-susceptible | pan-susceptible | S |
| 59 | TISS | - | 29.2 | 32.6 | pass | None | pan-susceptible | pan-susceptible | S |
| 60 | SPT | ++++ | 22.9 | 26.7 | pass | None | pan-susceptible | pan-susceptible | S |
| 61 | SPT | ++++ | 19.7 | 23.1 | pass | None | pan-susceptible | pan-susceptible | S |
| 62 | SPT | ++++ | 22.0 | 26.0 | pass | None | pan-susceptible | pan-susceptible | S |
| 63 | SPT | +++ | 25.6 | 28.6 | pass | None | pan-susceptible | pan-susceptible | S |
| 64 | SPT | ++++ | 20.3 | 23.5 | pass | None | pan-susceptible | pan-susceptible | S |
| 65 | SPT | + | 30.8 | 34.7 | n/a | n/a | n/a | pan-susceptible | S |
| 66 | SPT | ++ | 27.9 | 31.4 | pass | *rpoB* Leu511Pro  *katG* Ser315Thr  [*rpoB* Met434Val] | **RIF**  **INH** | **RIF**  **INH** | **MDR** |
| 67 | SPT | ++ | 23.1 | 26.4 | pass | None | pan-susceptible | pan-susceptible | S |
| 68 | SPT | ++ | 33.7 | undet | *rpoB, embB* | None | pan-susceptible EMB/RIF-cnd | pan-susceptible | S |
| 69 | SPT | ++++ | 24.0 | 27.6 | pass | [*rpoB* Thr663Ser] | pan-susceptible RIF-unk | pan-susceptible RIF-unk | S |
| 70 | SPT | ++++ | 20.2 | 23.4 | pass | None | pan-susceptible | pan-susceptible | S |
| 71 | SPT | ++ | 33.0 | 34.0 | n/a | n/a | n/a | pan-susceptible | S |
| 72 | SPT | +++ | 26.3 | 30.4 | *pncA* | *rpoB* Ser531Leu  *katG* Ser315Thr  *embB* Met306Ile  *rpsL* Lys43Arg  *rrs* A(1400)G  *gyrA* Asp94Asn  [*ethA* Leu478Arg] | **RIF**  **INH**  **EMB**  **SM**  **AMI/KAN**  **FQ**  **ETH-unk**  PZA-cnd** | **RIF**  **INH**  **EMB**  **SM**  **AMI/KAN**  **FQ**  **ETH-unk**  **PZA**** | **XDR** |

1. Specimen types include sputum (**SPT**), bronchoalveolar lavage (**BAL**), bronchial wash (**BRW**), pleural fluid (**PLF**), lung or lymph node (**TISS**), or unknown (**UNK**).
2. AFB smear results are abbreviated as numerous **(++++),** moderate **(+++),** few **(++),** rare as **(+),** and negative **(-).**
3. Real-time PCR assays were performed on the original specimens. Ct-values for *Mycobacterium tuberculosis* complex single-copy targets (**ExtRD9**) and multi-copy targets (**IS6110**) represent the average of two technical replicates.
4. “Pass” indicates that all targets met the minimum quality control (**QC**) requirements. Individual targets that fail to meet QC are indicated in parentheses and the corresponding antimicrobial susceptibility is listed as “could not determine (**cnd**).
5. High confidence resistance mutations detected. A full list can be found in **Supplementary Table 1**.
6. **RIF**, rifampin; **INH**, isoniazid; **PZA**, pyrazinamide; **EMB**, ethambutol; **FQ**, fluoroquinolones; **SM**, streptomycin; **KAN**, kanamycin; **AMI**, amikacin; **ETH**, ethionamide; -**res**, resistant; -**unk**; susceptibility unknown.
7. (*) Additional unknown mutations were detected with tNGS (performed on primary specimens) but were not detected with WGS (performed on cultured isolates). In this instance, a note will be included on the submitter report stating: “Targeted Next Generation Sequencing performed on primary specimens may detect population variants (unknown mutations) that are not identified with Whole Genome Sequencing. Culture-based testing should be performed for final susceptibility results.”
8. (**) PZA susceptibility was not determined via tNGS due to a failure to amplify the *pncA* locus. WGS identified a deletion in the *pncA* locus, indicative of pyrazinamide resistance.
