# Supplementary material for: Direct detection of drug-resistant Mycobacterium tuberculosis using targeted next generation sequencing
Source: Front Public Health. 2023 Jun 29;11:1206056. doi: 10.3389/fpubh.2023.1206056 (PMC10340549; doi:10.3389/fpubh.2023.1206056)
Supplement: Supplementary file 7 [file Table_7.DOCX]

**Table S7.** **Comparison of tNGS and WGS results for 55 MTBC-positive cultures** **with results obtained from WGS (performed on a purified MTBC isolate from the same patient**. 21 specimens were dual-positive for MTBC and *Mycobacterium avium* complex (MAC). Results indicate susceptibilities to 8 antimicrobials: rifampin, isoniazid, pyrazinamide, ethambutol, streptomycin, kanamycin/amikacin, fluoroquinolones, and ethionamide. Strains are considered “susceptible” for each drug unless otherwise indicated in the table below as resistant (**bold**), unknown (-unk), or could not determine (-cnd). Profiles are categorized as pan-susceptible (**S**), mono- or poly-resistant (**R**), multidrug resistant (**MDR**), pre-extensively drug resistant (**pre-XDR**), and extensively drug resistant (**XDR**).

| **ID** | **Real-time PCR results^1^** | | **tNGS Results** | | | **WGS Results** | **Profile** |
| --- | --- | --- | --- | --- | --- | --- | --- |
|  | **MTBC** | **MAC** | **QC^2^** | **High Confidence Mutations ^3^**  [Unknown mutations] | **Susceptibility Profiles^4^** | **Susceptibility Profiles^4^** |  |
| 73 | POS | NEG | Pass | None | Pan-susceptible | Pan-susceptible | S |
| 74 | POS | NEG | Pass | None | Pan-susceptible | Pan-susceptible | S |
| 75 | POS | NEG | Pass | [*katG* Phe540Ser] | INH-unk | INH-unk | S |
| 76 | POS | NEG | Pass | None | Pan-susceptible | Pan-susceptible | S |
| 77 | POS | NEG | Pass | *ethA* deletion | **ETH** | **ETH** | **R** |
| 78 | POS | NEG | Pass | *rpoB* Asp516Tyr  *katG* Ser315Thr  *rpsL* Lys43Arg  *gyrA* Asp94Ala | **RIF**  **INH**  **SM**  **FQ** | **RIF**  **INH**  **SM**  **FQ** | **Pre-XDR** |
| 79 | POS | NEG | Pass | *rpoB* His526Leu  *katG* Ser315Thr  *gyrA* Ala90Val;Asp94His  *ethA* insertion  [*pncA* Ala146Val]  [*embC-A* promoter C(-16)G] | **RIF**  **INH**  **FQ**  **ETH**  PZA-unk  EMB-unk | **RIF**  **INH**  **FQ**  **ETH**  PZA-unk  EMB-unk | **Pre-XDR** |
| 80 | POS | NEG | Pass | None | Pan-susceptible | Pan-susceptible | S |
| 81 | POS | NEG | Pass | None | Pan-susceptible | Pan-susceptible | S |
| 82 | POS | NEG | Pass | None | Pan-susceptible | Pan-susceptible | S |
| 83 | POS | NEG | Pass | None | Pan-susceptible | Pan-susceptible | S |
| 84 | POS | NEG | Pass | None | Pan-susceptible | Pan-susceptible | S |
| 85 | POS | NEG | Pass | None | Pan-susceptible | Pan-susceptible | S |
| 86 | POS | NEG | Pass | [*eis* promoter G(-81)T] | KAN/AMI-unk | KAN/AMI-unk | S |
| 87 | POS | NEG | Pass | None | Pan-susceptible | Pan-susceptible | S |
| 88 | POS | NEG | Pass | None | Pan-susceptible | Pan-susceptible | S |
| 89 | POS | NEG | Pass | None | Pan-susceptible | Pan-susceptible | S |
| 90 | POS | NEG | Pass | [*katG* Glu506Ala] | INH-unk | INH-unk | S |
| 91 | POS | NEG | Pass | None | Pan-susceptible | Pan-susceptible | S |
| 92 | POS | NEG | Pass | None | Pan-susceptible | Pan-susceptible | S |
| 93 | POS | NEG | Pass | *rpoB* Ser531Leu  *katG* Ser315Thr  *pncA* insertion  *embC-A* promoter C(-12)T  *rpsL* Lys88Arg | **RIF**  **INH**  **EMB**  **PZA**  **SM** | **RIF**  **INH**  **EMB**  **PZA**  **SM** | **MDR** |
| 94 | POS | NEG | Pass | *rpoB* Ser531Leu  *mabA* Leu203Leu  *embB* Met306Ile  *rpsL* Lys43Arg | **RIF**  **INH/ETH**  **EMB**  **SM** | **RIF**  **INH/ETH**  **EMB**  **SM** | **MDR** |
| 95 | POS | NEG | Pass | *mabA* promoter C(-15)T  *rpsL* Lys43Arg | **INH/ETH**  **SM** | **INH/ETH**  **SM** | **R** |
| 96 | POS | NEG | Pass | None | Pan-susceptible | Pan-susceptible | S |
| 97 | POS | NEG | Pass | None | Pan-susceptible | Pan-susceptible | S |
| 98 | POS | NEG | Pass | None | Pan-susceptible | Pan-susceptible | S |
| 99 | POS | NEG | Pass | None | Pan-susceptible | Pan-susceptible | S |
| 100 | POS | NEG | Pass | None | Pan-susceptible | Pan-susceptible | S |
| 101 | POS | NEG | Pass | None | Pan-susceptible | Pan-susceptible | S |
| 102 | POS | NEG | Pass | None | Pan-susceptible | Pan-susceptible | S |
| 103 | POS | NEG | Pass | None | Pan-susceptible | Pan-susceptible | S |
| 104 | POS | NEG | Pass | None | Pan-susceptible | pan-susceptible | S |
| 105 | POS | NEG | Pass | None | Pan-susceptible | pan-susceptible | S |
| 106 | POS | NEG | Pass | None | Pan-susceptible | pan-susceptible | S |
| 107 | POS | POS | Pass | None | Pan-susceptible | Pan-susceptible | S |
| 108 | POS | POS | Pass | None | Pan-susceptible | Pan-susceptible | S |
| 109 | POS | POS | Pass | None | Pan-susceptible | Pan-susceptible | S |
| 110 | POS | POS | Pass | *rpoB* Ser531Leu  *katG* Ser315Thr  *mabA-inhA* promoter C(-15)T  *embC-embA* C(-12)T  *rpsL* Lys88Arg  [*pncA* Ser104Arg]* | **RIF**  **INH**  **ETH**  **EMB**  **SM**  PZA-unk | **RIF**  **INH**  **ETH**  **EMB**  **SM**  PZA-unk | **MDR** |
| 111 | POS | POS | Pass | None | Pan-susceptible | Pan-susceptible | S |
| 112 | POS | POS | Pass | None | Pan-susceptible | Pan-susceptible | S |
| 113 | POS | POS | Pass | None | Pan-susceptible | Pan-susceptible | S |
| 114 | POS | POS | Pass | None | Pan-susceptible | Pan-susceptible | S |
| 115 | POS | POS | Pass | *katG* Ser315Thr | **INH** | **INH** | **R** |
| 116 | POS | POS | Pass | None | Pan-susceptible | Pan-susceptible  INH-cnd | S |
| 117 | POS | POS | Pass | None | Pan-susceptible | Pan-susceptible | S |
| 118 | POS | POS | Pass | None | Pan-susceptible | Pan-susceptible | S |
| 119 | POS | POS | Pass | None | Pan-susceptible | Pan-susceptible | S |
| 120 | POS | POS | ***embB*** | None | Pan-susceptible | Pan-susceptible | S |
| 121 | POS | POS | pass | None | Pan-susceptible | Unavailable^5^ | S |
| 122 | POS | POS | pass | [*rpoB* leu893Val]  [*embB* Gly65Ala] | RIF-unk  EMB-unk | Unavailable^5^ | S |
| 123 | POS | POS | ***gyrB*** | None | pan-susceptible (FLQ-cnd) | pan-susceptible | S |
| 124 | POS | POS | pass | [*rpoB* Glu747Lys] | Pan-susceptible  RIF-unk | Pan-susceptible  RIF-unk | S |
| 125 | POS | POS | pass | None | pan-susceptible | Pan-susceptible (INH-CND) | S |
| 126 | POS | POS | pass | None | pan-susceptible | pan-susceptible | S |
| 127 | POS | POS | ***embB*** | None | pan-susceptible (EMB-cnd) | Pan-susceptible | S |

1. Real-time PCR assays were performed on the original specimens. Positive (**POS**) or negative (**NEG**) results for MTBC (ExtRD9 and IS1160) and *Mycobacterium avium* complex (MAC, ITS target) are indicated.
2. “Pass” indicates that all targets met the minimum quality control (QC) requirements. Individual targets that fail to meet QC are indicated in parentheses and the corresponding antimicrobial susceptibility is listed as “could not determine” (-cnd).
3. High confidence resistance mutations detected. A full list can be found in **Supplementary Table 1**.
4. **RIF**, rifampin; **INH**, isoniazid; **PZA**, pyrazinamide; **EMB**, ethambutol; **FQ**, fluoroquinolones; **SM**, streptomycin; **KAN**, kanamycin; **AMI**, amikacin; **ETH**, ethionamide; -**res**, resistant; -**unk**; susceptibility unknown.
5. High-quality sample was not available for WGS analysis.
6. Specimens #31-34 and #48-55 were performed as part of a prospective study.
